# A cross‐sectional study assessing modifications to the delivery of a multi‐component implementation strategy (the Get Outside, Get Active program) to improve child physical activity in early childhood education and care services

**DOI:** 10.1002/hpja.920

**Published:** 2024-08-26

**Authors:** Luke Giles, Nicole Pearson, Hannah Lamont, Alice Grady, Sze Lin Yoong

**Affiliations:** ^1^ Hunter New England Population Health Wallsend New South Wales Australia; ^2^ School of Medicine and Public Health, College of Health, Medicine and Wellbeing University of Newcastle Callaghan New South Wales Australia; ^3^ Hunter Medical Research Institute New Lambton Heights New South Wales Australia; ^4^ Global Obesity Centre, Institute for Health Transformation, School of Health and Social Development, Faculty of Health Deakin University Geelong Victoria Australia

**Keywords:** adaptations, early childhood education, implementation, modifications, physical activity

## Abstract

**Issue Addressed:**

The Get Outside, Get Active (GOGA) program is a randomised controlled trial which tested the impact of a multi‐component implementation strategy to support early childhood education and care (ECEC) services to replace indoor‐only free play with indoor–outdoor‐free play. This cross‐sectional study aims to describe the extent and nature of modifications made to implementation strategies and Behaviour Change Techniques (BCTs) using the Framework for Reporting Adaptations and Modifications to Evidence‐based Implementation Strategies (FRAME‐IS) and to describe the fidelity of BCT delivery throughout GOGA.

**Methods:**

An audit of records was undertaken throughout the intervention delivery period in the intervention arm. GOGA included 14 standard BCTs within six implementation strategies. Modifications and BCT delivery were recorded by Health Promotion Officers via project records. Modifications were categorised according to the FRAME‐IS. BCT delivery was recorded using a checklist.

**Results:**

Forty‐four ECEC services received the GOGA program. Overall, 60 modifications were recorded. According to FRAME‐IS categories, most modifications related to: content; format; pragmatic or practical considerations; tailoring/tweaking/refining in nature; fidelity was inconsistent; the goal was to increase the acceptability, appropriateness, or feasibility of the implementation effort; the rationale was at the practitioner level; and were unplanned/reactive. Overall, 96.4% of standard BCTs were delivered as intended.

**Conclusions:**

GOGA was delivered with high fidelity to protocol as indicated by the level of BCT delivery. This article details a thorough approach to documenting modifications and provides guidance for future studies.

**So What?:**

This article contributes to the emerging evidence regarding documentation of adaptations and modifications to public health implementation interventions.

## 1 INTRODUCTION

Physical activity (PA) in young children is beneficial for child health, physical development, and preventing chronic disease.^1^, ^2^, ^3^, ^4^, ^5^, ^6^ In Australia, national PA guidelines for children aged 3–5 years recommend daily PA of ‘at least 180 minutes spent in a variety of physical activities, of which at least 60 minutes is energetic play, spread throughout the day; more is better’.^7^ Population‐based surveys indicate that only 61% of Australian children aged 2–5 years are meeting national PA recommendations.^8^ Interventions to increase the amount of time that children spend in PA in the early childhood education and care (ECEC) setting are recommended by the World Health Organisation.^9^, ^10^ ECEC services are ideal settings for such interventions as they provide access to the majority of children aged 3–5 years for a significant proportion of the day.^11^

Increasing outdoor‐free play opportunities in ECEC services is associated with increased child moderate‐to‐vigorous physical activity (MVPA).^12^ Outdoor‐free play is defined as when children can decide what they want to do, where they want to be and with whom they interact while in the outdoor environment.^13^ Guidelines recommend that 60–90 min of outdoor play is provided daily in ECEC services.^14^, ^15^, ^16^ Randomised controlled trials (RCTs) undertaken by the research team suggest that amending the way in which outdoor‐free play opportunities are structured, such as allowing children to move freely between the indoor and outdoor environments during free play times or increasing the frequency of outdoor‐free play opportunities, can increase child MVPA.^12^, ^17^

Despite the benefits of increasing outdoor‐free play in ECEC, evidence suggests that children are not provided with sufficient opportunities. For example, a systematic review by Truelove and colleagues reported that the average duration of outdoor play was 45 minutes.^18^ The Get Outside, Get Active (GOGA) trial is a RCT which sought to test the impact of a multi‐component implementation strategy supporting ECEC services to replace periods of indoor‐only free play with indoor–outdoor‐free play, ultimately increasing both the amount and frequency of opportunities for children to access outdoor play environments. Implementation strategies are ‘methods or techniques used to enhance the adoption, implementation, and sustainability of a clinical program or practice’,^19^ and include strategies such as ‘Identify and prepare champions’ and ‘Distribute educational materials’. The GOGA program also included the use of Behaviour Change Techniques (BCTs) to address barriers to the provision of outdoor‐free play. BCTs are the ‘observable, replicable, and irreducible component of an intervention designed to alter or redirect causal processes that regulate behaviour’^20^ and include BCTs such as ‘Goal setting (behaviour)’ and ‘Action planning’. The GOGA trial was delivered to ECEC services within the Hunter New England region of New South Wales (NSW).

Adaptation of an evidence‐based practice (EBP) has been defined as the deliberate alteration of the design or delivery of the EBP to improve its fit in a given context,^22^ including to fit the needs of participants such as ECEC services. Modifications ‘can include adaptations, which are planned or purposeful changes to the design or delivery of an intervention, but they can also include unintentional deviations from the interventions as originally designed’.^23^ Documenting modifications systematically is recommended to better understand the specific processes and strategies that lead to successful implementation.^24^ Understanding modifications also provides contextual and process data that can support the interpretation of study findings and can help identify which intervention components are important to retain for future scale‐up.^22^, ^25^ Although a number of frameworks such as the Framework for Reporting Adaptations and Modifications to Evidence‐based Implementation Strategies (FRAME‐IS) have been developed to enable researchers and practitioners to record adaptations and modifications,^22^, ^23^, ^26^, ^27^ documentation and reporting of adaptations and modifications has not been undertaken consistently.^22^ Although some studies have documented adaptations using the FRAME‐IS,^28^, ^29^ few have published systematic documentation of modifications using this framework.

### 1.1 Objectives

Therefore, this study sought to:

1. describe the extent and nature of modifications made to implementation strategies and BCTs (i.e. discrete BCTs or multiple BCTs where whole implementation strategies were impacted) throughout GOGA using the FRAME‐IS and
2. describe the fidelity of BCT delivery (considered as the core components) throughout GOGA.

## 2 METHODS

### 2.1 Study design

This cross‐sectional study reports on an internal audit of modifications made to implementation strategies and BCTs, and the delivery of BCTs, within the intervention group of the GOGA RCT.

#### 2.1.1 Core components of the GOGA trial

The GOGA trial methods are described in detail elsewhere.^30^ Eligible services within the Hunter New England region of NSW were identified via a computer assisted telephone interview, with a further recruitment call made by the research team. Block randomisation was used to allocate services to the intervention and control groups. GOGA was designed to target barriers to increasing outdoor‐free play opportunities in ECEC services. The Behaviour Change Wheel^31^ and Theoretical Domains Framework^32^ were used to identify key barriers to implementation. Using a systematic mapping process, BCTs were selected to address barriers and support implementation of indoor–outdoor‐free play routines. These were delivered via six implementation strategies selected from the Expert Recommendations for Implementing Change (ERIC) taxonomy.^21^ Implementation strategies were delivered over seven planned intervention contacts via video and telephone calls over six months by Health Promotion Officers (HPOs). The role of HPOs is to facilitate communities to improve their health through the planning, implementation and evaluation of health promotion policies and projects.^33^, ^34^

Implementation strategies included:

- identify and prepare champions,
- distribute educational materials,
- conduct educational outreach visit(s),
- develop a formal implementation blueprint,
- provide local centralised technical assistance, and
- performance feedback and review.

A description of these implementation strategies as they relate to GOGA is available within the protocol.^30^

Protocols for each service contact were developed which included the key purpose of the contact, and content to guide the delivery of the implementation strategy and BCTs. These protocols were designed to allow for a level of flexibility in the delivery of GOGA to align with service needs. HPOs were instructed to use their discretion as a facilitator and balance planned intervention delivery with the needs and priorities of services, while recording any adjustments to the delivery of intervention contacts. Intervention delivery occurred during a period where COVID‐19 restrictions had recently been lifted for the region, and the research team anticipated this may also impact on the need for intervention modifications.

A total of 18 BCTs were planned to be delivered as part of GOGA (see Appendix S1). Fourteen of these were standard BCTs which were to be delivered to each ECEC service, and some of which were delivered during multiple intervention contacts. There were 36 planned instances of standard BCT delivery throughout the intervention per service (11 of the 14 standard BCTs were delivered during multiple intervention contacts). Six optional BCTs could be delivered to services that were identified as requiring further support. There were 35 potential instances for optional BCTs to be delivered across multiple intervention contacts per service.

Ethics approval was provided by Hunter New England (HNE) Human Research Ethics Committee (HREC) (reference no 2019/ETH12353), University of Newcastle HREC (reference no H‐2008‐0343) and Deakin University HREC (ref 2023‐062).

### 2.2 Data collection

Data were collected via the HPOs delivering the intervention. The recording of modifications and BCT delivery was based on methods consistent with other studies and amended to suit the aims of this study.^35^, ^36^, ^37^

#### 2.2.1 Modifications and BCT delivery

Where any change to the delivery of intervention contacts, implementation strategies or BCTs were made, a modification was recorded according to the FRAME‐IS. The FRAME‐IS was selected as the most suitable framework as it is designed to record modifications to implementation strategies and to facilitate assessment of the core components and functions of implementation strategies.^22^ Some adjustments were made to the FRAME‐IS to align with GOGA – for example, removing some categories within the ‘what is the goal’ construct that were not relevant, such as ‘Improve sustainability of the EBP’. In addition, the ‘how’ and ‘impact’ constructs from the Triple Aim Quality Enhancement Research Initiative Adapted Stirman Adaptation (TAQERIASA) framework^27^ were used to record these elements of modifications. Additionally, brief notes were recorded to provide additional context to modifications. Data were recorded by HPOs after the completion of intervention contacts.

Members of the research team regularly discussed the recording of modifications. Data entry guidelines were developed to standardise recording, with consensus processes undertaken for recording any new modifications. Quality assurance checks were undertaken throughout intervention delivery to ensure consistency of data entry.

The delivery of BCTs during GOGA was recorded immediately post‐intervention contact by HPOs within the project‐specific monitoring database in REDCap, using a check box to indicate delivery.

### 2.3 Data analysis

Data analysis was undertaken in Microsoft Excel. Descriptive statistics (n, %) were used to describe modifications and BCT delivery. Modifications were categorised by the implementation strategy during which they occurred, and FRAME‐IS and TAQERIASA framework constructs and categories.

## 3 RESULTS

Forty‐five services were randomised to receive the GOGA implementation strategies, and 43 were randomised to the control group (not included in this study as they did not receive the implementation strategy). For the intervention group, one service was found to be ineligible after randomisation, 14 services withdrew from the trial during intervention delivery, and 30 services completed the intervention. Reasons for services withdrawing from the intervention included staffing issues, time constraints, competing priorities, and change of ownership. The results from the 44 eligible services are reported.

### 3.1 Modifications

In total, 60 modifications were recorded, with these modifications occurring in 27 of the 44 (61.4%) ECEC services. Twenty‐one services received all BCTs as per protocol. Four services where a modification was recorded received the intervention as per protocol, but the modification related to the provision of additional support or change in protocol that did not impact BCT delivery. The number of modifications per service ranged from one (*n* = 14 services) to six (*n* = 1 service). Six services accounted for 28 (46.7%) of the modifications that were recorded.

The number and percentage of modifications made, recorded by the implementation strategy during which the modification occurred, are reported in Appendix S2. Most modifications were made during the ‘Conduct educational outreach visit(s)’ implementation strategy (*n* = 29).

Table 1 reports the modifications made during GOGA by the constructs and categories of the FRAME‐IS and parts of the TAQERIASA framework.

**TABLE 1:** Modifications made during GOGA reported according to the FRAME‐IS and TAQERIASA framework constructs and categories.

Construct	Category	Number of modifications	Percentage of modifications for construct category (%)
What is modified?^a^	Content	44	72.1
	Context	17	27.9
Context^a^, ^b^	Format	10	58.8
	Setting	7	41.2
How or on what basis was this change made^c^	Based on pragmatic/practical consideration	50	83.3
	Based on feedback or suggestions	9	15.0
	Other	1	1.7
What is the nature of the content, evaluation or training modification?^a^	Tailoring/tweaking/refining	37	61.7
	Adding elements	1	1.7
	Removing/skipping elements	12	20.0
	Lengthening/extending	2	3.3
	Reordering of intervention modules or segments	4	6.7
	Spreading	1	1.7
	Integrating another strategy into the implementation strategy in primary use	1	1.7
	Repeating elements or modules of the implementation strategy	1	1.7
	Departing from the implementation strategy (‘drift’) followed by a return to strategy within the implementation encounter	1	1.7
Relationship to fidelity/core elements^a^	Fidelity consistent/Core elements or functions preserved	23	38.3
	Fidelity inconsistent/Core elements or functions changed	37	61.7
What is the goal?^a^	Increase adoption of the EBP	1	1.7
	Increase the acceptability, appropriateness, or feasibility of the implementation effort	57	95.0
	Increase health equity or decrease disparities in EBP delivery	1	1.7
	Other	1	1.7
What is the level of the rationale for modification?^a^	Organisational level	16	26.7
	Implementer level	15	25.0
	Practitioner level	29	48.3
When is the modification initiated?^a^	Implementation phase	60	100
Is modification planned?^a^	Planned/Proactive (proactive adaptation)	3	5.0
	Planned/Reactive (reactive adaptation)	16	26.7
	Unplanned/Reactive (modification)	41	68.3
Who participates in the decision to modify?^a^	Program Leader, Manager, or Administrator	11	10.8
	Implementer or implementation strategy expert	38	37.3
	Clinician(s) or teacher(s) who are being asked to use the EBP being implemented	53	52.0
How widespread is the modification?^a^	Patients or other recipients that share a particular characteristic	1	1.7
	Individual clinician or teacher charged with implementing the EBP	15	25.0
	Organisation	41	68.3
	Specific implementer/facilitator	3	5.0
Impact: What are (subjective) short‐term results of the adaptation?^c^	Positive (e.g. more participation, improved delivery, improved outcomes, reduced costs)	14	23.3
	Negative (e.g. less participation, reduced delivery, reduced outcomes, increased costs)	15	25.0
	No real impact	31	51.7

*Note*: FRAME‐IS categories with zero modifications have not been included in this table – for all FRAME‐IS categories, refer to Miller et al.^22^ Modifications could be coded to multiple categories within a construct, therefore some construct totals exceed the total number of modifications recorded, and where this occurs the percentage has been calculated using the totals for the construct.

^a^ FRAME‐IS constructs and categories.

^b^ Context construct is only applicable when ‘context’ is selected for the ‘What is modified’ construct, therefore totals for this construct are lower.

^c^ TAQERIASA framework constructs and categories.

The most frequently recorded modifications are described in Table 2. These modifications account for 34 (56.7%) of all modifications.

**TABLE 2:** Description of the most common modifications recorded during GOGA.

Type of modification	Frequency (*n*, % of all modifications)	Number of services impacted by this modification (*n*, %)	Implementation strategies impacted	BCTs impacted
Change to timing of goal setting (i.e. service did not set a draft goal specifying how the service would increase indoor–outdoor‐free play during the initial intervention contact)	16, 26.7%	16, 36.4%	‐ Conduct educational outreach visit(s) (intervention contact: Educational meeting)	1.1 Goal setting (behaviour) (BCT was still delivered to 15 services, generally later in the intervention and thus at a lower dosage)
Non‐delivery of implementation strategies	10, 16.7%	5, 11.4%	‐ Conduct educational outreach visit(s) (intervention contacts: Educator information presentation & Consultation process) ‐ Provide local centralised technical assistance ‐ Performance feedback and review	All BCTs included within the implementation strategies impacted (see Appendix S1 for BCTs that were planned to be delivered during each implementation strategy)
Change to modality (i.e. HPOs using alternate modes of contact for intervention delivery (e.g. face‐to‐face instead of virtual meetings; providing resources via hand‐delivery)))	8, 13.3%	5, 11.4%	‐ Distribute educational materials ‐ Conduct educational outreach visit(s) (intervention contacts: Educator information presentation & Consultation process) ‐ Provide local centralised technical assistance ‐ Performance feedback and review	Nil (BCTs were still delivered via alternate modality)

In total, 35 (58.3%) modifications resulted in a BCT or implementation strategy not being delivered at all or at a reduced dose. Of these modifications, 15 (42.9%) remained unresolved (i.e. undelivered) at the end of the intervention. For modifications that were resolved, resolution generally occurred by completing a BCT that was not delivered during the intended intervention contact at a later intervention contact.

Modifications were recorded for 66.7% of services that completed GOGA. Of the 14 services that withdrew from the intervention arm, six withdrew prior to commencing intervention delivery. For services that withdrew after commencing intervention delivery, seven (87.5%) recorded a modification, with 11 modifications recorded in total prior to their withdrawal.

### 3.2 The extent to which BCTs were delivered according to protocol

Almost two‐thirds of services (63.6%, 28/44) received all standard BCTs at least once, and half of all services (22/44) received the full dose of all standard BCTs at all planned occasions. The optional BCTs were delivered to 75% (33/44) of services.

In total, 96.4% of the standard BCTs were delivered as planned, and 95.6% of the optional BCTs were delivered when indicated. The fidelity of delivery of standard BCTs is described in Table 3.

**TABLE 3:** Fidelity of delivery of standard BCTs during the implementation of GOGA.

Standard BCTs	Fidelity of delivery
1.1 Goal setting (behaviour)	92.9%
1.4 Action planning	100.0%
1.5 Review behaviour goal(s)	95.7%
2.2 Feedback on behaviour	90.3%
2.3 Self‐monitoring of behaviour	95.7%
2.7 Feedback on outcome(s) of behaviour	90.3%
3.1 Social support (unspecified)	98.2%
3.2 Social support (practical)	100.0%
4.1 Instruction on how to perform the behaviour	95.7%
5.1 Information about health consequences	95.7%
5.3 Information about social & environmental consequences	95.7%
6.3 Information about others' approval	100.0%
9.1 Credible source	95.7%
12.1 Restructuring the physical environment	100.0%
Total standard BCT delivery	96.4%

## 4 DISCUSSION

Modifications were widespread, with 61.4% of services making at least one modification. Modifications were typically minor in nature, reflected by the high percentage recorded as ‘Tailoring/tweaking/refining’ (61.7%). Adherence to the intervention protocol was high, with 60 modifications recorded in total out of 226 scheduled intervention contacts and approximately 1240 occasions of standard BCT delivery.

Modifications did not impede overall BCT delivery. The active components of the intervention were still delivered, with over 96% of standard BCTs delivered as planned, indicating the intervention had good service fit. The core components of the intervention were often delivered regardless, as demonstrated by two of the most frequent modifications being related to a change in timing or modality. This shows the importance of specifying key aims for intervention contacts, including BCTs, to allow for the core components to be delivered at another time or in another way if necessary.

Most modifications were made in response to specific service needs. For example, the large number of services that did not set a goal during the first educational meeting (*n* = 16) is reflective of the collaborative practice of ECEC services,^38^ with many indicating a preference to consult with other staff first. All but one of these services set a goal at a later stage. This is important given the positive association between goal setting and PA outcomes.^39^, ^40^, ^41^

As 75% of modifications were categorised by HPOs as positive or having no real impact, this suggests they did not significantly affect intervention delivery and were unlikely to negatively impact on the main trial outcomes from the perspective of the HPO. By comparison, a physical activity intervention in secondary schools recorded 20 modifications during the 24‐month intervention with 24 schools, with 79% of modifications having a positive impact.^36^ The higher number of modifications in our study was due to recording at an individual service level, whereas the schools study reported on modifications made at the intervention level which impacted on all schools in the intervention arm.

Some major modifications occurred, such as services not receiving BCTs and implementation strategies as per protocol. For example, the ‘Performance feedback and review’ implementation strategy was not delivered to three services, resulting in the BCTs ‘Feedback on behaviour’ and ‘Feedback on outcome(s) of behaviour’ not being received. Removal of this implementation strategy may have had a small impact on the effect of the intervention for these services.^42^ The reasons for non‐delivery typically related to broader contextual factors, for example ECEC staffing challenges, time constraints, and other priorities. This is similar to the reasons that services did not receive intervention components in a previous trial by members of the research team.^35^ Although attempts were made to deliver all strategies, this reflects real‐world research where it may not always be possible to deliver full interventions as per protocol. Although attempts were made to ensure flexibility with intervention contacts, it is likely that future interventions need to consider the minimum dose required for effect.

The findings highlight some considerations for any future iteration of GOGA, such as addressing challenges related to time pressures, staffing considerations, and the consultative practice of ECEC services. These issues were reflected in some modifications related to non‐delivery and change to modality of intervention components. The findings of this study should be reviewed alongside other trial results to inform potential adaptations and decision making related to the scale up or optimisation of GOGA.^36^ Any adaptations should also consider possible impacts on intervention efficacy.^43^

The higher rate of modifications among services that withdrew from GOGA (87.5% of services that commenced intervention delivery) compared with services that completed GOGA (66.7% of services) could suggest that modifications are an indicator of poor intervention or service fit due to challenging circumstances at a service, competing priorities, or a service being at increased risk of withdrawal. It is likely that the number of modifications during GOGA may have been even higher if these services did not withdraw, due to the potential for additional modifications during the undelivered intervention contacts.

A previous systematic review of adaptations of 42 evidence‐based public health interventions by Escoffery and colleagues found no articles described adaptation or modification in the ECEC setting,^44^ and a review by Marshall et al. identified five obesity prevention interventions in ECEC which were all cultural adaptations of interventions.^45^ The authors are aware of two other studies describing adaptation to public health interventions in the ECEC setting – the adaptation of a school‐based intervention for ECEC services,^46^ and a study reporting adaptations to an intervention for scale up^28^; however, these do not report modifications. These articles reflect the emerging nature of the evidence regarding the documentation of modifications to public health implementation interventions, including for interventions in the ECEC setting, and therefore this article provides an important contribution to the evidence‐base.

### 4.1 Context

Throughout the implementation of GOGA, several contextual factors impacted on intervention delivery. These included the ongoing impacts of COVID‐19 including staffing pressures within the ECEC sector due to isolation procedures, broader workforce challenges (e.g. staff shortages), and extreme weather events experienced by services.

### 4.2 Strengths and limitations

Strengths of this study include the use of a standardised framework to record modifications to a multi‐component implementation strategy (FRAME‐IS), which enables consistency in reporting data.^43^ To our knowledge, this is one of the first studies reporting on modifications – rather than adaptations – to an intervention using the FRAME‐IS. Other strengths include the addition of further elements from the TAQERIASA framework^27^ to provide additional context, and the use of a database to record implementation activities at the time of delivery by HPOs, rather than less reliable methods such as self‐report.^47^

Limitations include some modifications being reviewed and re‐coded retrospectively as some FRAME‐IS elements were not accurately captured when the data collection instrument was established. There is the potential for some misinterpretation of the modification when recoding since this was completed several months after the modification occurred. Additionally, the process of recording modifications does not capture modifications that may have been made at the service level. For example, services may have modified intervention components without the knowledge of the research team.

## 5 CONCLUSIONS

Modifications made during the GOGA trial were typically minor, with a neutral to positive affect on intervention delivery. Furthermore, BCTs were delivered with high fidelity to protocol.

This article details a thorough approach to documenting modifications and provides guidance for similar future studies. This article contributes to building the emerging evidence regarding documentation of adaptations and modifications to public health implementation interventions and the application of the FRAME‐IS.

## FUNDING INFORMATION

This work was supported by a National Health and Medical Research Council (NHMRC) Partnership grant (APP1170042). NHMRC had no role in the design of this study and did not have any role during its execution, analyses, interpretation of the data or the decision to publish results. Cash and in‐kind support were provided by Hunter New England Population Health (HNEPH) and Centre for Population Health (CPH) at the NSW Ministry of Health. Representatives from HNEPH and CPH sit on the trial advisory group as part of co‐production and community engagement processes, and HNEPH staff are co‐authors on this manuscript. SY was supported by a Heart Foundation Future Leader Fellowship (106654). AG receives salary support via a Heart Foundation Postdoctoral Fellowship (102518).

## CONFLICT OF INTEREST STATEMENT

The authors declare that they have no competing interests.

## ETHICS STATEMENT

In accordance with the Declaration of Helsinki, ethics approval has been provided by Hunter New England (HNE) Human Research Ethics Committee (HREC) (reference no 2019/ETH12353), University of Newcastle HREC (reference no H‐2008‐0343) and Deakin University HREC (ref 2023‐062).

## Supporting information


**Appendix S1:** BCTs mapped by implementation strategy and intervention contact per service.
**Appendix S2:** number and percentage of modifications made during each implementation strategy.

## Data Availability

The data that support the findings of this study are available on request from the corresponding author. The data are not publicly available due to privacy or ethical restrictions.
