# Supplementary material for: A cross‐sectional study assessing modifications to the delivery of a multi‐component implementation strategy (the Get Outside, Get Active program) to improve child physical activity in early childhood education and care services
Source: Health Promot J Austr. 2024 Aug 26;36(2):e920. doi: 10.1002/hpja.920 (PMC11806367; doi:10.1002/hpja.920)
Supplement: Supplementary file 1 — Appendix S1: BCTs mapped by implementation strategy and intervention contact per service. Appendix S2: number and percentage of modifications made during each implementation strategy. [file HPJA-36-0-s001.docx]

**Appendices**

**Appendix 1: BCTs mapped by implementation strategy and intervention contact per service.**

| **BCTs** | **Implementation strategy (intervention contact)** | | | | | | | | |
| --- | --- | --- | --- | --- | --- | --- | --- | --- | --- |
|  | Identify and prepare champions  (N/A) | Distribute educational materials  (N/A) | Conduct educational outreach visit(s) (Educational meeting) | Conduct educational outreach visit(s)  (Educator information presentation) | Conduct educational outreach visit(s)  (Consultation process) | Develop a formal implementation blueprint  (Action plan meeting) | Provide local centralised technical assistance  (Support meeting 1) | Performance feedback and review  (Audit & feedback meeting) | Provide local centralised technical assistance  (Support meeting 2) |
| ***Standard BCTs*** | | | | | | | | | |
| 1.1 Goal setting (behaviour) ^1^ |  |  | 🗸 |  |  | 🗸 |  |  |  |
| 1.4 Action planning ^1^ |  |  | 🗸 |  |  | 🗸 |  |  |  |
| 1.5 Review behaviour goal(s) |  |  |  |  |  |  | 🗸 | 🗸 | 🗸 |
| 2.2 Feedback on behaviour |  |  |  |  |  |  |  | 🗸 |  |
| 2.3 Self-monitoring of behaviour |  |  |  |  |  |  | 🗸 | 🗸 | 🗸 |
| 2.7 Feedback on outcome(s) of behaviour |  |  |  |  |  |  |  | 🗸 |  |
| 3.1 Social support (unspecified) | 🗸 |  | 🗸 | 🗸 |  | 🗸 | 🗸 | 🗸 | 🗸 |
| 3.2 Social support (practical) | 🗸 | 🗸 |  |  |  |  |  |  |  |
| 4.1 Instruction on how to perform the behaviour |  | 🗸 | 🗸 | 🗸 |  |  |  |  |  |
| 5.1 Information about health consequences |  | 🗸 | 🗸 | 🗸 |  |  |  |  |  |
| 5.3 Information about social & environmental consequences |  | 🗸 | 🗸 | 🗸 |  |  |  |  |  |
| 6.3 Information about others’ approval | 🗸 | 🗸 |  |  |  |  |  |  |  |
| 9.1 Credible source |  | 🗸 | 🗸 | 🗸 |  |  |  |  |  |
| 12.1 Restructuring the physical environment |  |  | 🗸 |  |  |  |  |  |  |
| ***Optional BCTs*** | | | | | | | | | |
| 1.1 Goal setting (behaviour) ^1^ |  |  |  |  | 🗸 |  | 🗸 | 🗸 | 🗸 |
| 1.2 Problem solving |  |  | 🗸 | 🗸 | 🗸 | 🗸 | 🗸 | 🗸 | 🗸 |
| 1.4 Action planning ^1^ |  |  |  |  | 🗸 |  | 🗸 | 🗸 | 🗸 |
| 1.6 Discrepancy between current behaviour & goal | 🗸 |  |  |  |  |  | 🗸 | 🗸 | 🗸 |
| 15.1 Verbal persuasion about capability | 🗸 |  | 🗸 | 🗸 | 🗸 | 🗸 | 🗸 | 🗸 | 🗸 |
| 15.3 Focus on past success | 🗸 |  | 🗸 | 🗸 | 🗸 | 🗸 | 🗸 | 🗸 | 🗸 |

^1^ BCT is listed within both the standard and option BCTs categories, based on how it was intended to be delivered within various implementation strategies.

**Appendix 2: number and percentage of modifications made during each implementation strategy.**

| **Implementation strategy (number of standard BCTs planned to be delivered during implementation strategy)** | **Number of modifications made** | **Percentage of all modifications made (%)** |
| --- | --- | --- |
| Identify and prepare champions (3) | 0 | 0.0 |
| Distribute educational materials (6) | 6 | 10.0 |
| Conduct educational outreach visit(s) (8) | 29 | 48.3 |
| Develop a formal implementation blueprint (3) | 0 | 0.0 |
| Provide local centralised technical assistance (3) | 12 | 20.0 |
| Performance feedback and review (5) | 10 | 16.7 |
| Other (e.g. service paused participation in GOGA; additional contact delivered via service visit) (N/A) | 3 | 5.0 |
| Total | 60 | 100.0 |
